# Anti-Seizure Activity of 1-Adamantane Carboxylic Acid in Common Experimental Seizure Models: Role of Benzodiazepine-GABA_A_ Receptors

**DOI:** 10.52547/ibj.25.3.213

**Published:** 2021-03-03

**Authors:** Elham Ghanbari, Hakimeh Gavzan, Bahar Khoshkroodian, Mohammad Sayyah

**Affiliations:** 1 Department of Physiology and Pharmacology, Pasteur Institute of Iran, Tehran, Iran;; 2 Department of Basic Sciences, Faculty of Veterinary Medicine, Amol University of Special Modern Technologies, Amol, Iran

**Keywords:** Anticonvulsants, Flumazenil, Pentylenetetrazole

## Abstract

**Background:**

Despite introduction of modern antiepileptic drugs, 30% of epileptic patients are still drug resistant. Remarkable three-dimensional spatial structure of AdCA, yet the simplicity of the molecule, makes AdCA a promising lead compound.

**Methods:**

Sedative/motor impairment and 24-h mortality rate of AdCA were determined in mice. Impact of AdCA on (1) threshold and occurrence of clonic seizures induced by PTZ in mice, (2) incidence of tonic seizures induced by MES in mice, and (3) incidence of generalized seizures and duration of evoked afterdischarges in amygdala-kindled rats, were determined. The role of benzodiazepine receptors in the AdCA effect on clonic seizure threshold was also assessed.

**Results:**

AdCA showed sedative effect (TD_50 _= 224.5 [190.2-289.9] mg/kg). LD_50 _= 805.5 (715.2–988.1) mg/kg was obtained for AdCA. The compound increased PTZ seizure threshold from 180 mg/kg (*p *< 0.05) and also inhibited the incidence of clonic seizures (ED_50 _= 256.3 [107.4-417.3] mg/kg). AdCA also decreased afterdischarge duration (*p *< 0.01) and the incidence of generalized seizures (ED_50 _< 50 mg/kg) in the kindled rats. However, AdCA did not protect mice against tonic seizures induced by MES. The benzodiazepine receptor antagonist flumazenil prevented the increase of seizure threshold by AdCA.

**Conclusion:**

AdCA possesses anticonvulsant activity in kindling and PTZ models through the activation of benzodiazepine/GABA_A_ receptors with acceptable therapeutic index.

## Introduction

Epilepsy is a neurologic disease with 1% global prevalence. Regardless of the development of innovative anticonvulsant medications, drug therapy is not accompanied by the successful control of seizures in one-third of the epileptic patients^[^^1^^]^. Therefore, finding efficient antiepileptic drugs with the least adverse effect is still demanded.

**Fig. 1:**
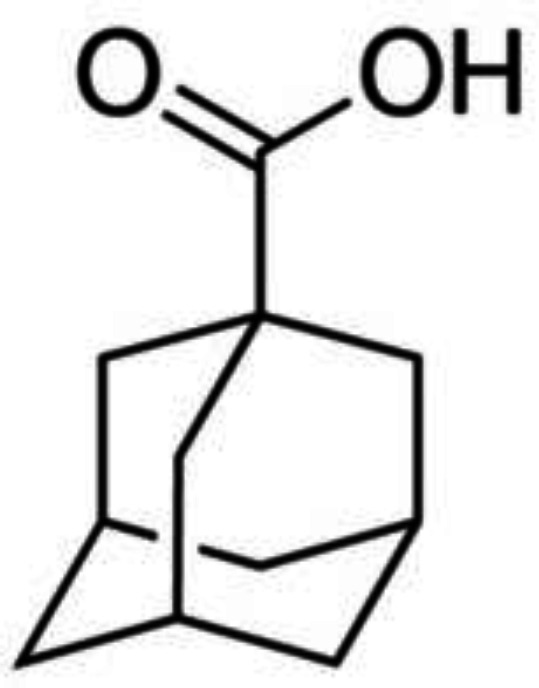
**Chemical structure of** **AdCA**

Using bioinformatics tools and pharmacophore drug design, we have previously introduced several compounds with potential anticonvulsant activity^[^^2^^]^. Though all candidate molecules had adequate safety, a few compounds were effective in experimental seizure models^[^^2^^]^. One of the candidate molecules was AdCA (Fig. 1), which did not show anti-seizure activity in PTZ and MES tests up to 1 mM after intracerebro-ventricular administration. However, there is an old short report that indicates the anticonvulsant activity of AdCA in MES and PTZ models after i.p. administration to mice^[^^3^^]^. We examined the effect of non-sedative doses of AdCA but did not find any anticonvulsant activity^[^^2^^]^. However, in the report of Fridman *et al.*^[^^3^^]^, it has not mentioned whether any sedative effect is detected at the anticonvulsant doses. Meanwhile, AdCA has been a molecule of interest for medicinal chemists in the last 50 years and widely used for the improvement of the pharmacodynamics and pharmacokinetics of drugs and biologically active compounds^[^^4^^]^. 

The present study was aimed to do a full screen of anticonvulsant, sedative and lethal effects of AdCA in the most commonly used screening seizure tests in experimental animals. MES test is an animal model of primary generalized tonic seizures and detects substances inhibiting distribution of seizure activity. On the other hand, PTZ test is an animal model of generalized myoclonic seizures and identifies compounds increasing seizure threshold^[^^5^^]^. Electrical kindling is the experimental model of focal seizures with secondary generalization^[^^6^^]^ with close resemblance to TLE^[^^7^^]^, the most common form of epilepsy and the most frequent drug-resistant one in adults^[^^8^^]^. Kindling model of epilepsy is broadly used to determine the efficacy of the candidate molecules for the treatment of TLE^[^^6^^]^. Thus, the efficacy of AdCA against PTZ, MES, and amygdala-kindled seizures was assessed in the current study.

## MATERIALS AND METHODS

**Animal**s 

NMRI mice (adult male, 20–26 g, n = 335) and Wistar rats (adult male, 280–320 g, n = 32) were provided by the Pasteur Institute of Iran (Tehran). Animals were kept in standard polypropylene boxes in an animal room under a 12:12 h light/dark cycle (07.00 a.m. to 07.00 p.m.) and regulated temperature (23 ± 2.0 °C). They had free access to rodent chow and drinking water. All the experiments were performed during light cycle (10:00-16:00). In order to get used to the laboratory environment, animals were always taken to the laboratory one hour before the start of the experiments.

**Drugs**

AdCA, flumazenil, PTZ, and DMSO were purchased from Sigma-Aldrich, Germany. PTZ and flumazenil solutions were prepared freshly in NaCl 0.9% andsesame oil, respectively. AdCA was dissolved in DMSO and then reached the desired concentration by sesame oil (DMSO:sesame oil ratio was 30:70). 

**Assessment of neurotoxicity and lethality**

Before assessing the anticonvulsant activity, neurotoxic and lethal doses of AdCA were determined. Lethality was determined by the i.p. injection of the solvent (DMSO 30% in sesame, 10 ml/kg) and AdCA 500, 600, 700, 900, and 1500 mg/kg to six groups of mice with 10 mice in each group. The number of deaths was recorded till 24 h after injection. Sedation and motor impairment were assessed in mice by rotarod test according to the method described previously^[^^9^^]^. Mice were trained to be able to walk on a horizontal rotating rod (3.5 cm diameter and 15 rpm speed) for a period of consecutive 120 s. The motor performance of mice was checked on the day of experiments before injections. Then the solvent (DMSO 30% in sesame, 10 ml/kg) and AdCA 100, 180, 230, and 300 mg/kg were injected i.p. to five groups of mice with 10 mice in each group. After 30 min, the mice were given three opportunities to remain on the rod for a period of 120 consecutive seconds. The endurance time on the rod and the number of animals, which fell off the rod within 60 s, were recorded. 

**Evaluation of the rate of clonic seizures induced by PTZ**

Clonic seizures were provoked in mice by i.p. injection of PTZ 60 mg/kg. GC was considered as the endpoint. GC is regarded as clonus of whole body with loss of righting reflex. If no GC occurred during a 30-min period of observation, the animals were considered protected. In order to determine the time course of AdCA anti-seizure effect, AdCA 300 mg/kg or the solvent was injected, i.p., to mice (each one to 3 groups with 10 mice in each group). The incidence of GC was recorded at 30, 90, and 180 min after the injection. In order to obtain dose-response data, AdCA 0 (the solvent), 180, 240, 300, and 400 mg/kg were injected i.p., to five groups of mice (10 mice in each group). The incidence of GC was recorded after 30 min.

**Determination of the threshold of clonic seizures induced by PTZ**

PTZ 10 mg/ml was infused into the lateral tail vein of freely moving mice at a constant rate of 100 µl/min according to the method established before^[^^10^^]^. The volume of PTZ solution required for the induction of GC was recorded. Then the amount of PTZ (mg) per mouse body weight (kg) was calculated and considered as the threshold of seizure for that animal. The maximum volume of intravenous infusion to each mouse was 200 µl. If a mouse did not show GC up to 200 µl infusion, it was excluded from the study. Four groups of mice (with eight mice in each) received AdCA 0 (the solvent), 100, 180, and 300 mg/kg. Threshold of clonic seizures was determined 30 min thereafter. Seizure threshold was also verified in a group of mice with no injection. In order to assess the possible interaction of AdCA with GABA_A_/BZD receptors, five groups of mice (eight mice in each) were allocated. Two groups received flumazenil (10 mg/kg, i.p.) or its solvent, and seizure threshold was measured after 30 min. The dose and time effect of flumazenil was selected based on our previous study^[^^9^^]^. In the two other groups, mice were pretreated with flumazenil (10 mg/kg). After 20 min, AdCA 180 and/or 300 mg/kg was/were injected to the mice, and after 30 min, seizure threshold was measured. The fifth group was control group in which the mice were pretreated with the solvent of flumazenil. After 20 min, the solvent of AdCA was injected, and after 30 min, seizure threshold was measured. 

**Determination of the incidence of tonic seizures induced by MES**

Tonic seizure was induced in mice by electroconvulsive shock (50 mA, 50 Hz, sine wave, 0.2 sec duration) via ear clip electrodes using a stimulator apparatus (MES9312, SATA). Tonic seizure is characterized by a tonic extension in the hind limbs of mice and defined as HLTE. If HLTE did not happen for three min, mice were considered protected. AdCA 0 (the solvent), 300, and 400 mg/kg, i.p., were injected to three groups of mice (10 mice in each group). The incidence of HLTE was recorded after 30 min.

**Amygdala kindling**

Amygdala-kindled rats were prepared according to the procedure described previously^[^^11^^]^. Rats were stereotaxically implanted with bipolar stimulating and monopolar recording electrodes in the basolateral amygdala (coordinates: A, −2.5 mm from bregma; L, 4.8 mm from bregma; V, 7.3 mm from dura) of the right hemisphere. The rats were given one week to recover. The procedure of rapid amygdala kindling was then started. AD threshold of amygdala was determined for each rat by a 5-s, 50-Hz monophasic square-pulse stimulus of 1 msec per pulse. The stimulus current started from 50 μA. If no AD was recorded, then the current increased progressively in increments of 50 μA every five min until at least five s AD was recorded. This current was considered as the AD threshold for that animal. Then each animal was stimulated at AD threshold by a 12 trains/day schedule with a 5-min interval between each train. Behavioral seizures were scored based on Racine classification as stage 1 (S1), facial clonus; stage 2 (S2), head nodding; stage 3 (S3, focal seizures), unilateral forelimb clonus; stage 4 (S4), rearing and bilateral forelimb clonus; stage 5 (S5, generalized seizures), rearing, loss of balance and falling^[^^12^^]^. Animals were stimulated every day until three sequential stage five seizures were observed. These animals were considered fully kindled. Four groups of the kindled rats (eight rats in each group) were pretreated with AdCA 0 (the solvent), 50, 100, and 180 mg/kg, i.p. Duration of AD, duration of S5, and the incidence of S5 in each animal were measured after 30 min.

**Statistical analysis**

SPSS for Windows software (version 22) was used for statistical analysis. The normality of the quantitative data, including endurance time on the rotarod, seizure threshold, duration of AD, and duration of S5, was assessed by Shapiro-Wilk test. The data had a normal distribution and were presented as mean ± SEM. Therefore, they were analyzed by parametric test, i.e. one-way analysis of variance (ANOVA) and Tukey’s post hoc test. The incidence of clonic seizures in PTZ test, tonic seizures in MES test, and generalized seizures (S5) in the kindled rats were analyzed by Fisher's exact probability test. The log-probit method^[^^13^^]^ was used to calculate the ED_50_, LD_50_, and TD_50_ of AdCA and the corresponding 95% confidence limits. The PI and the TI of AdCA were calculated by dividing the obtained TD_50_ and LD_50_ by the obtained ED_50_, respectively. The difference between the groups with *p* value less than 0.05 was considered statistically significant.

**Ethical statement**

The above-mentioned treatment protocols and experiments accomplished based on the guidelines of Institutional Animals Ethics Committee of Pasteur Institute of Iran (ethical code: IR.PII.REC.1394.48) and EU Directive 2010/63/EU. 

## RESULTS

**Neurotoxicity and lethality of AdCA**

**Fig. 2:**
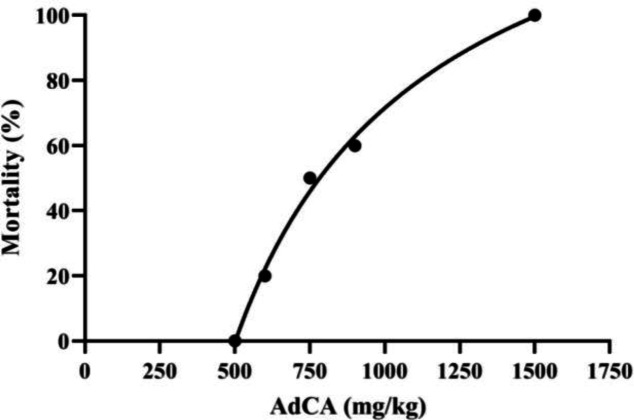
**D** **ose** **-** **mortality curve of AdCA in mice**

**Fig. 3:**
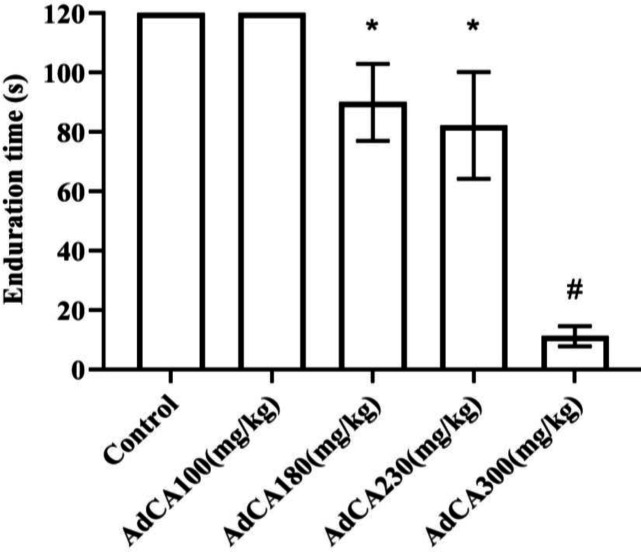
Effect of AdCA on rotarod performance of mice. *p < 0.05 and #p < 0.001, compared to the control group

Figure 2 demonstrates dose-mortality curve of AdCA. LD_50_ value of 805.5 (715.2–988.1) mg/kg was obtained for AdCA in mice. As shown in Figure 3, the solvent of AdCA had no effect on rotarod performance 

of mice. Administration of AdCA at the concentration of 180 mg/kg or higher decreased endurance time on the rotarod (*p *< 0.05). The sedative/motor impairment activity was dose-dependent, and TD_50_ value of 224.5 (190.2-289.9) mg/kg was obtained for AdCA. 

**Anti-seizure effect of AdCA in PTZ model**

**Fig. 4:**
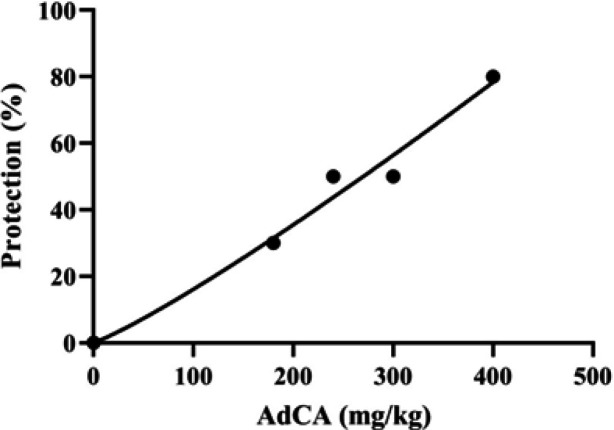
Dose-response curve of AdCA anticonvulsant activity against clonic seizures induced by PTZ in mice

**Fig. 5:**
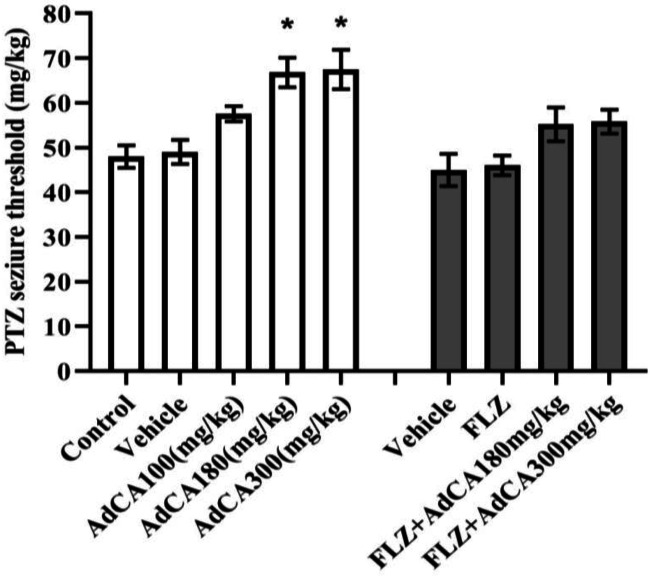
Effect of AdCA on the threshold of seizures induced by PTZ in mice. ^*^p <0.05 compared to the control and vehicle groups. FLZ, flumazenil

The anti-seizure effect of AdCA against clonic seizures induced by i.p. PTZ was statistically significant (*p *< 0.05) at 30 min after injection (Table 1). Dose-response curve of AdCA anticonvulsant activity against clonic seizures is presented in Figure 4. The ED_50_ value of 256.3 (107.4-417.3) mg/kg was obtained for AdCA. PI value of 0.87 and TI value of 3.58 were obtained for AdCA. AdCA also significantly increased the clonic seizure threshold (Fig. 5). Pretreatment of mice with flumazenil prevented the elevation of the seizure threshold by both dose 180 and 300 mg/kg of AdCA (Fig. 5). 

**Effect of AdCA in MES model**

At the tolerable dose 300 mg/kg of AdCA, no significant anti-seizure activity was observed against tonic seizures induced by MES (Table 2). 

**Anti-seizure effect of AdCA in amygdala-kindled rats**

**Fig. 6:**
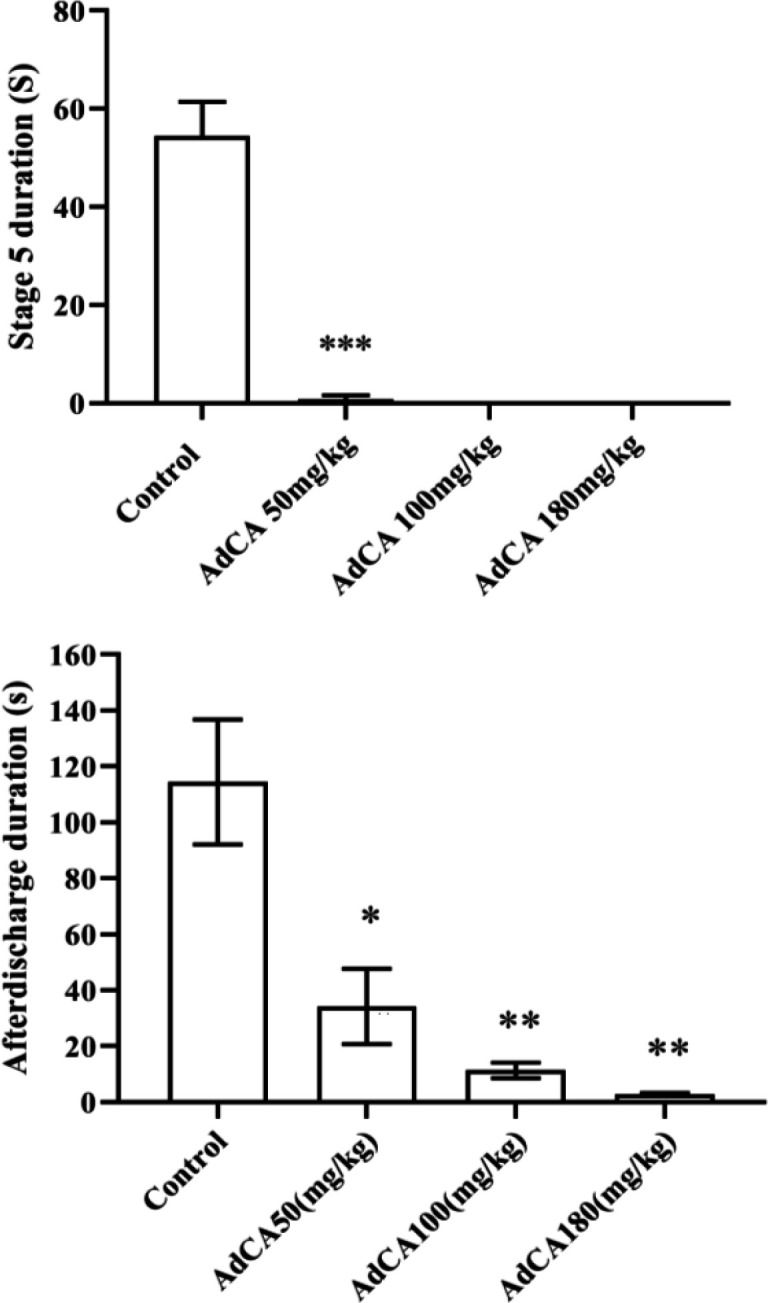
**Effect of** AdCA on the duration of generalized seizures and amygdala-evoked ADs in rats. ^*^p < 0.05, ^**^p < 0.01, and  ^***^p < 0.001 compared to the control group

AdCA significantly suppressed the incidence of generalized kindled seizures in rats, from the dose 50 mg/kg (Table 2), as well as the duration of generalized seizures, and ADs evoked in amygdala (Fig. 6). 

**Table 1:** Time course of AdCA protective effects in PTZ test

**Treatment (mg/kg)**	**Time interval between AdCA and PTZ injection (min)**	**Number of mice with seizure/total mice**
Solvent (Control)	30, 90, 180	10/10
AdCA (300)	30	5/10^*^
AdCA (300)	90	8/10
AdCA (300)	180	10/10

Data were analyzed by Fisher's exact probability test. ^*^p < 0.05 compared to the control group

## DISCUSSION

We have found in the present study that AdCA has anticonvulsant activity in both PTZ model of clonic seizures and kindling model of complex partial seizures. The anticonvulsant effect of AdCA is mediated (in part) through benzodiazepine/GABA_A_ receptors. AdCA showed acceptable anti-seizure and therapeutic indices in mice.

AdCA is a lipophilic hydrocarbon, which has been used in recent 50 years as a backbone platform for the synthesis of several drugs from different pharmacological categories. Among these AdCA-based drugs, the aminoadamantane-based medicines, amantadine and memantine, are the most famous medicines approved for the treatment of neurologic diseases^[^^4^^]^. The unique chemical structure of AdCA accounts for its use in design and modification of drugs to change the pharmacokinetics and/or pharmacodynamics properties of a compound^[^^14^^]^. Nevertheless, AdCA itself also possesses pharmacologic activity. It has previously been reported that AdCA has anticonvulsant activity^[^^3^^]^. Moreover, it is a potent and specific inhibitor of the enzyme, ceramide kinase, and can, therefore, control the cell signaling^[^^15^^]^. We found in the present study that AdCA was able to decrease incidence and susceptibility to clonic seizure induced by PTZ. AdCA also showed sedative effect; the anti-seizure effect of AdCA appeared at the sedative doses. This finding is in line with our previous study in which the non-sedative doses of AdCA did not show anticonvulsant activity in PTZ and MES models^[^^2^^]^. Results of our study showed that the margin between sedative and anticonvulsant doses of AdCA was narrow in the PTZ model as PI value of 0.87 was obtained for AdCA. In the present study, AdCA did not show any anti-seizure effect against tonic seizures induced by MES, even at the highest sedative dose (300 mg/kg). This observation is in contrast to the report of Fridman *et al.*^[^^3^^]^ who reported 300 mg/kg of ED_50_ value for AdCA in MES test. 

We observed that the benzodiazepine receptor antagonist flumazenil entirely inhibited the anti-seizure effect of AdCA. Therefore, it is suggested that the anti-seizure activity of AdCA in PTZ model is mediated through the modulation of the benzodiazepine allosteric site of the GABA_A_ receptors. To the best of our knowledge, this is the first report regarding the modulation of benzodiazepine allosteric site by AdCA. It is well known that PTZ-induced seizures are prevented by drugs, such as benzodiazepines, that enhance GABA_A_ receptor-mediated inhibitory transmission^[^^16^^]^ and/or decline transient calcium currents, such as ethosuximide^[^^17^^]^. Therefore, it seems that AdCA is also able to block calcium channels. In line with this suggestion, electrophysiological evidence indicates that the adamantane-derived GABA shows anticonvulsant activity and reduces neuronal calcium current^[^^18^^]^. In addition, drugs that block glutamatergic excitation mediated by n-methyl-D-aspartate receptors, such as felbamate, have anticonvulsant activity against PTZ-induced seizures^[^^16^^]^. The adamantane derivatives, amantadine and memantine, blocks n-methyl-D-aspartate receptors^[^^4^^]^. Thus, AdCA inhibits PTZ-induced clonic seizures by several mechanisms.

In the current study, AdCA could inhibit amygdala-kindled seizures. It is precious that this inhibitory effect appeared at the non-sedative doses of AdCA.

**Table 2:** Effect of 1-adamantane carboxylic acid on the seizure incidence in kindling and MES models of seizures

**Seizure model**	**Treatment (mg/kg)**	**Number of animals with seizure/total number of animals**	**ED**_50_ ** (mg/kg)**
MES (tonic seizures)	Solvent (control)	10/10	-
	AdCA (300)	8/10	
	AdCA (400)	2/10^**^	
Kindling (complex partial seizures)	Solvent (control)	8/8	<50
	AdCA (50)	1/8^**^	
	AdCA (100)	0/8^**^	
	AdCA (180)	0/58^**^	

Data were analyzed by Fisher’s exact probability test. ^**^p < 0.01 compare to the control group.

Several mechanisms are suggested for the inhibition of kindled seizures, including activation of GABA receptors and modulation of ion channels^[^^19^^-^^21^^]^. We found in this study that GABA_A_ receptors were contributed to the anticonvulsant activity of AdCA against PTZ seizures. Therefore, it is possible that this mode of action is implicated in the anticonvulsant effect of AdCA against kindled seizures. This proposal needs to be examined in future studies. Drugs with efficacy against kindled seizures have the potential to be effective against partial seizures and TLE^[^^6^^]^. Therefore, AdCA and its derivatives are expected to be effective against TLE, as the most common form of drug-resistant epilepsy. It is promising that the low and non-sedative doses of AdCA exert potent anticonvulsant effect in the kindling model. This finding makes AdCA and/or its derivatives worthy for further comprehensive evaluations for the treatment of drug-resistant epilepsy. 

In conclusion, AdCA shows anticonvulsant activity against clonic seizures in the PTZ model through modulation of benzodiazepine binding site on GABA_A_ receptors. The valuable finding of the present study might be that AdCA at low and non-sedative doses inhibits complex partial seizures in the amygdala-kindling model. These findings uncover more facets of this appreciated molecule.
